# Crocin-I Protects Against High-Fat Diet-Induced Obesity *via* Modulation of Gut Microbiota and Intestinal Inflammation in Mice

**DOI:** 10.3389/fphar.2022.894089

**Published:** 2022-08-11

**Authors:** Xiaoxian Xie, Mengya Zhang, Lei Sun, Ting Wang, Zhengyan Zhu, Ruonan Shu, Fengchun Wu, Zezhi Li

**Affiliations:** ^1^ College of Biotechnology and Bioengineering, Zhejiang University of Technology, Hangzhou, China; ^2^ Department of Psychiatry, The Affiliated Brain Hospital of Guangzhou Medical University, Guangzhou, China; ^3^ Guangdong Engineering Technology Research Center for Translational Medicine of Mental Disorders, Guangzhou, China

**Keywords:** crocin-i, gut microbiota, inflammation, lipid metabolism, obesity

## Abstract

Crocin-I can regulate physiological changes in the human body by altering inflammation and microbial composition. Gut microbiota are also involved in modulating the pathophysiology of obesity. However, crocin-I’s effect on obesity and the mechanism underlying its effects on gut microbiota and inflammation remain poorly understood. Here, high-fat diet (HFD) -induced obese mice were administrated crocin-I (20 mg/kg/day) for 10 weeks using an oral gavage (HFD-C20 group). HFD-C20, HFD, and Normal chow (NC) groups were compared. The fat content, colon tissue inflammatory cytokine levels, gut microbiota, and short-chain fatty acids (SCFAs) levels were measured. We show that crocin-I reduced body weight and liver weight and improved glucose resistance in HFD-induced mice, and reduced the lipid accumulation in the liver. Strikingly, crocin-I alleviated intestinal microbial disorders and decreased the F/B ratio and the abundance of *Proteobacteria* in HFD-induced obese mice. Crocin-I also rescued the decrease in the levels of SCFAs and repaired altered intestinal barrier functioning and intestinal inflammation in HFD-induced obese mice. These findings indicate that crocin-I may inhibit obesity by modulating the composition of gut microbiota and intestinal inflammation.

## Graphical Abstract

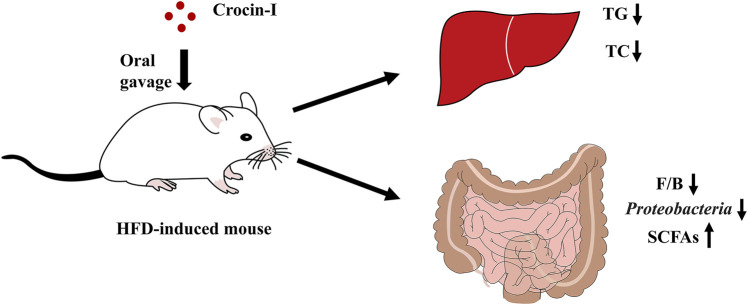

## Introduction

Obesity has become a worldwide health issue characterized by abnormal or excessive lipid accumulation (Carron et al., 2020). Obesity is also an inducing factor of dyslipidemia, cancer, hypertension, diabetes, cardiovascular complications, and several psychiatric disorders, all of which are associated with increased medical and economic burdens (Kopelman, 2000; Opel et al., 2015).

The current mainstays of obesity treatment are diet and physical exercise. However, obese patients who have failed lifestyle treatments may need further interventions, including medication (Ryder et al., 2018). Although great progress has been made in our understanding of how obesity develops, the adverse effects of many anti-drugs have also become more obvious as time goes on (Kang and Park, 2012). In recent years, a variety of functional foods have been reported to prevent and alleviate the physiology and pathology of obesity by directly regulating the expression of obesity-related genes or fixing intestinal microbiota imbalances (Rayalam et al., 2008; Xie et al., 2019).

*Crocus sativus* L., which is commonly known as saffron, has been widely cultivated in many countries for thousands of years because of its pro-circulatory and anti-stasis functions. Crocin is a series of ester glycosides of crocetin and is the main active ingredient of saffron (Kalalinia et al., 2018). Crocin’s bioavailability varies and it can be used by intestinal microbiota in the large intestine, it can also regulate physiological changes in the body by affecting microbial compositions (Xie et al., 2019). An increasing body of evidence suggests that gut microbiota are essential for the development of obesity and treating obesity by modulating the composition of gut microbiota has gradually become more mainstream (Torres-Fuentes et al., 2017). For example, the ratio of *Firmicutes*/*Bacteroidetes* (F/B) has been reported to be increased in obese animals and crowds (Grigor’eva, 2020; Magne et al., 2020; Duan et al., 2021). In addition, high-fat diets (HFDs) can regulate the activity of short-chain fatty acid (SCFA)-producing bacteria, thereby changing butyrate, propionate, and acetate content in the body (Zhang et al., 2019). A growing body of evidence has demonstrated that consuming some functional foods and extracts can regulate intestinal microbiota compositions and have anti-obesity effects (Jiao et al., 2019; Zhou et al., 2020). However, any possible anti-obesity effects of crocin-I (a major member of the crocin family that is comprised of crocin I-IV) (Xie et al., 2019) in HFD-induced obese mice, and/or how gut microbiota may change after supplementation with crocin-I, remain unknown.

Here, we attempt to explore whether crocin-I supplementation can mitigate obesity in HFD-induced obese mice. We also confirm the potential relationship between gut microbiota and the metabolic benefits of crocin-I, which may help deepen our understanding of the mechanism underlying saffron phytochemicals in preventing or treating metabolic diseases.

## Materials and Methods

### Animal and Experiment Procedure

Four-week-old male C57BL/6J mice (*n* = 40) were bought from the Shanghai SLAC Laboratory Animal Company Limited (Shanghai, China). During the experiment, the mice were kept in a well-maintained and hygienic environment (at a 12 h light/12 h dark cycle, with 55 ± 5% humidity and 23 ± 1°C) with free access to water and food. The mice were randomly divided into two groups after one week of acclimation (NC group: Normal chow diet, *n* = 20; HFD-induced group: high-fat diet, *n* = 20). The HFD-induced obese mice received a pellet diet (Rodent Diet D12492; Research Diets, United States), where fat provided 60% of calories, carbohydrates provided 20%, and protein provided 20%. Following an 8-week induction (*p* < 0.01 relative to the NC group), the HFD-induced obese mice (*n* = 20) were randomly divided into two groups: treatment with crocin-I (20 mg/kg/day; HFD-C20), or vehicle alone (HFD group). Both groups were continuously fed with an HFD for the next 10 weeks.

Mice in the HFD-C20 group were supplied with 20 mg/kg of crocin-I daily for 10 weeks using oral gavage. The NC group and HFD group received the same amount of RO water as the crocin-I group. Body weight was recorded once a week, and mean food consumption was measured three times a week for 10 consecutive weeks starting at the 8th week.

Serum was obtained from blood (4°C, 5000 rpm, 10 min) and stored at −80°C. The colon, cecal contents and fecal samples, and liver, kidney, and epididymal white adipose tissues (WAT) were stored at −80°C.

This animal study protocol was reviewed and approved by the Zhejiang University of Technology Ethics of Animal Experiments committee (Permission no. SYXK (Zhejiang) 2017-0001). The experimental design for the study is shown in Supplementary Figure S1.

### Crocin-I Preparation and Dosage Information

Crocin- I was isolated as previously described (Xie et al., 2019). The HFD-C20 group was dosed with crocin-I (20 mg/kg/day bodyweight; human equivalent dose estimated to be ∼150 mg/day for a 70 kg-adult) *via* gavage. The dose was selected based on our previous study, which showed crocin-I’s hepatoprotective effects in C57BL/6J mice (Xie et al., 2019).

### Biochemical Parameter Measurements

The liver tissues from each mouse were isolated and homogenized with nine volumes of cold PBS. These supernatants were collected using centrifugation (12,000 rpm, 10 min). The protein concentration was measured using a commercial BCA protein kit (Beyotime Institute of Biotechnology, Shanghai, China). TG and TC levels were detected using Wako Labassay kits (LabAssay^TM^ ALP, Wako, Japan).

### Histological Analysis

The accumulation and droplet distribution of lipids in hepatic cells were measured using hematoxylin and eosin (H&E), and Oil Red O staining (*n* = 4 per group), as previously described in Xie et al. (2019). Briefly, we soaked fresh liver tissue slices in 4% paraformaldehyde fixation solution and placed them at room temperature for 24 h. The tissues were snap-frozen and cut into 5 μm-sections on glass slides. Next, the slides were incubated with Oil Red O and H&E, respectively. After a series of processes, the tissues (4 μm section) were stained with H&E based on a standard method, while the colon tissue was treated with H&E and Alcian blue-periodic acid Schiff (AB-PAS) solution, respectively. Pathological changes were observed under a light microscope (Olympus, Tokyo, Japan) and were analyzed with Image-Pro Plus 6.0 (Media Cybernetics Inc., United States).

### Intraperitoneal Glucose Tolerance Test (IPGTT)

Before IPGTT, the mice were fasted overnight for approximately 16 h. The fasting blood glucose was tested, which was recorded as t = 0 min. The mice were then intraperitoneally injected with 10% D-glucose (2 g/kg body weight). Blood glucose levels were tested 15, 30, 60, 90, and 120 min after glucose injection by placing a small drop of blood on a new test strip and recording the measurements. The area under the blood glucose curve was calculated.

### Short-Chain Fatty Acids (SCFAs) Analysis

Fecal pellets were collected after the crocin- I or HFD treatments. Fecal water was prepared by homogenizing collected pellets with 1 mM 2-ethylbutyric acid in 15% phosphate acid for 8 min using a vortex mixer. The supernatants were obtained after 12,000 rpm centrifugation for 10 min at 4°C, they were then syringe filtered with 0.22 μm filters, and transferred to 250 μL inserts (Agilent Technologies, Palo Alto, CA). As soon as the fecal supernatants reached a final concentration of 1 mM, they were stored in 2 ml GC vials. SCFAs were detected with gas chromatography using a Trace 1310 gas chromatograph (Thermo Fisher Scientific, Waltham, MA), coupled with a flame ionization detector (Thermo). A data assay was performed with gas chromatography–mass spectrometry (GC-MS) solution software (4.45).

### Real-Time Quantitative Polymerase Chain Reaction (RT-qPCR)

Total RNA was extracted from liver, epididymal adipose, and colon tissues using TRIzol reagents (Invitrogen Co., Carlsbad, CA, United States). RT-qPCR was performed using methods that have previously been described in Xie et al. (2021). Expression levels of selected genes were normalized to *β*-actin gene levels. The specific primers are listed in Supplementary Table S1. The levels of relative expression were detected using 2^−ΔΔCT^.

### DNA Extraction and 16S rRNA Gene Sequencing

Microbial genomic DNA (gDNA) was extracted from thawed cecal contents using a QIAamp DNA stool Mini Kit (QIAGEN, Carlsbad, CA), and was then quantified via UV spectroscopy. The V3-V4 region in 16S rRNA gene of the bacteria was PCR-amplified from microbial genome DNA using specific primers (forward primer: 5′-ACT​CCT​ACG​GGA​GGC​AGC​AG-3′; reverse primer: 5′GGACTACHVGGGTW-TCTAAT-3′). QIIME (vision 1.6.0) was performed to detect alterations in the gut microbiota.

### Statistical Analysis

All of the data are expressed as means ± SEM. One-way Analysis of variance (ANOVA) in GraphPad Prism (version 7.0). Tuckey’s multiple comparison tests were used when multiple groups were being compared. A *p* value <0.05 was considered statistically significant.

## Results

### Crocin-I Alleviates the Growth Phenotype and Biochemical Index in HFD-Induced Obese Mice

**FIGURE 1:**
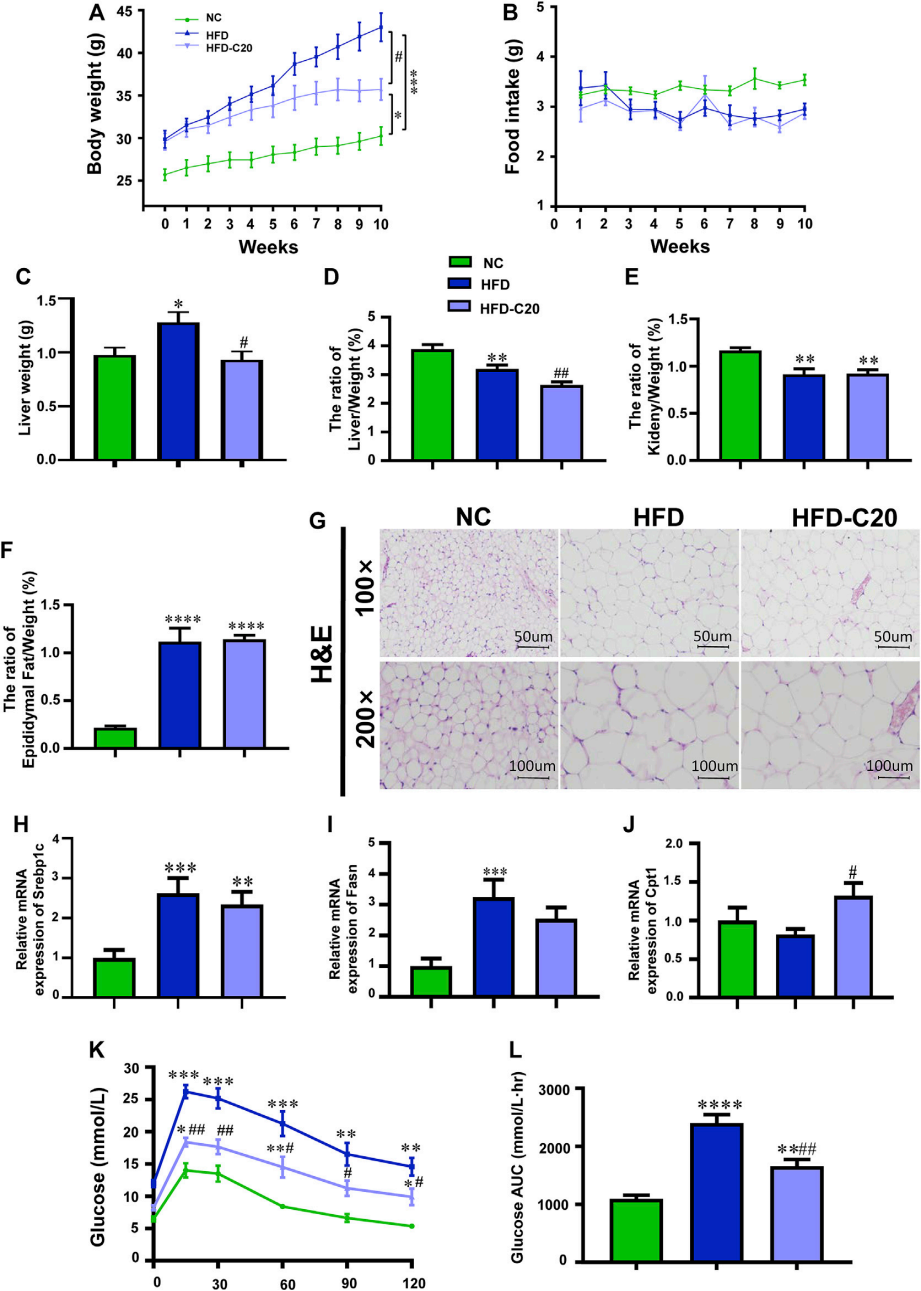
Crocin-I alleviates growth phenotypes and biochemical index of HFD-induced obese mice. **(A)** Body weight; **(B)** food intake; **(C)** liver weight; **(D)** the ratio of liver/weight, **(E)** the ratio of kidney/weight, and **(F)** the ratio of epididymal fat/weight (*n* = 8); **(G)** lipid accumulation in WAT revealed by H&E staining. **(H–J)** The transcription levels of genes related to fatty acid synthesis in WAT. **(K)** Blood glucose value and (L) Area under the curve (AUC). *n* = 8 per group. Values represent mean ± SEM. *p < 0.05 and **p < 0.01, compared with control group; ^#^ *p* < 0.05 and ^##^ *p <* 0.01, compared with HFD group.

Between the 3rd and 9th weeks, mice that were fed with an HFD showed increased weights compared to control mice. However, mice that then had 7 weeks of crocin-I treatment showed decreased weights compared to mice fed with HFD alone (Figure 1A; Supplementary Figure S2). At the end of the experiment, the HFD group had a 42.5% increase in body weight compared to the NC group, while crocin-I treatment decreased the body weight of the HFD group by 15.4% (Figure 1A). Additionally, no significant alterations in average food intake (g/d/mouse) were detected between the HFD and HFD-C20 groups (Figure 1B), which indicates that the influence of crocin-I on body weight was not associated with changes in food consumption. Furthermore, although liver weights increased significantly in the HFD group compared with the control group, this trend was reversed with the crocin-I treatment (Figure 1C). However, the ratio of liver to body weight (Figure 1D) and the ratio of the kidney to body weight (Figure 1E) decreased significantly in the HFD group relative to the NC group (Figure 1D) because increases in body weight were largely determined by increases in epididymal fat (Figure 1F). We also found that kidney weight and epididymal fat were not affected by crocin-I treatment (Figures 1E, F). H&E staining increased adipose cell volume in the WAT of HFD-induced obese mice, but crocin-I administration did not reverse this trend (Figure 1G). We then analyzed the mRNA expression of lipid-related genes in the WAT. The results show that mRNA expression of sterol regulatory element binding protein 1c (*Srebp1c*) and fatty acid synthase (*Fasn*) were increased in the HFD group compared with the NC group, whereas, crocin-I treatment did not reverse their changes in mRNA levels (Figures 1H,I). Additionally, a high-fat diet did not significantly influence mRNA expression of carnitine palmitoyltransferase 1 (*Cpt1*) in the HFD group, but crocin-I significantly increased mRNA expression of *Cpt1* (Figure 1J). Taken together, these results suggest that crocin can possibly relieve obesity by regulating the phenotypes of the liver. Furthermore, IPGTT indicated that, in contrast with the NC group, the HFD group had increased blood glucose concentrations, and the HFD group had a higher area under the curve (Figures 1K,L). Although crocin-I treatment significantly reduced blood glucose concentrations in the HFD group, it did not restore them to the levels seen in the controls.

### Crocin-I Alleviates High-Fat Diet-Induced Lipid Accumulation in the Liver

**FIGURE 2:**
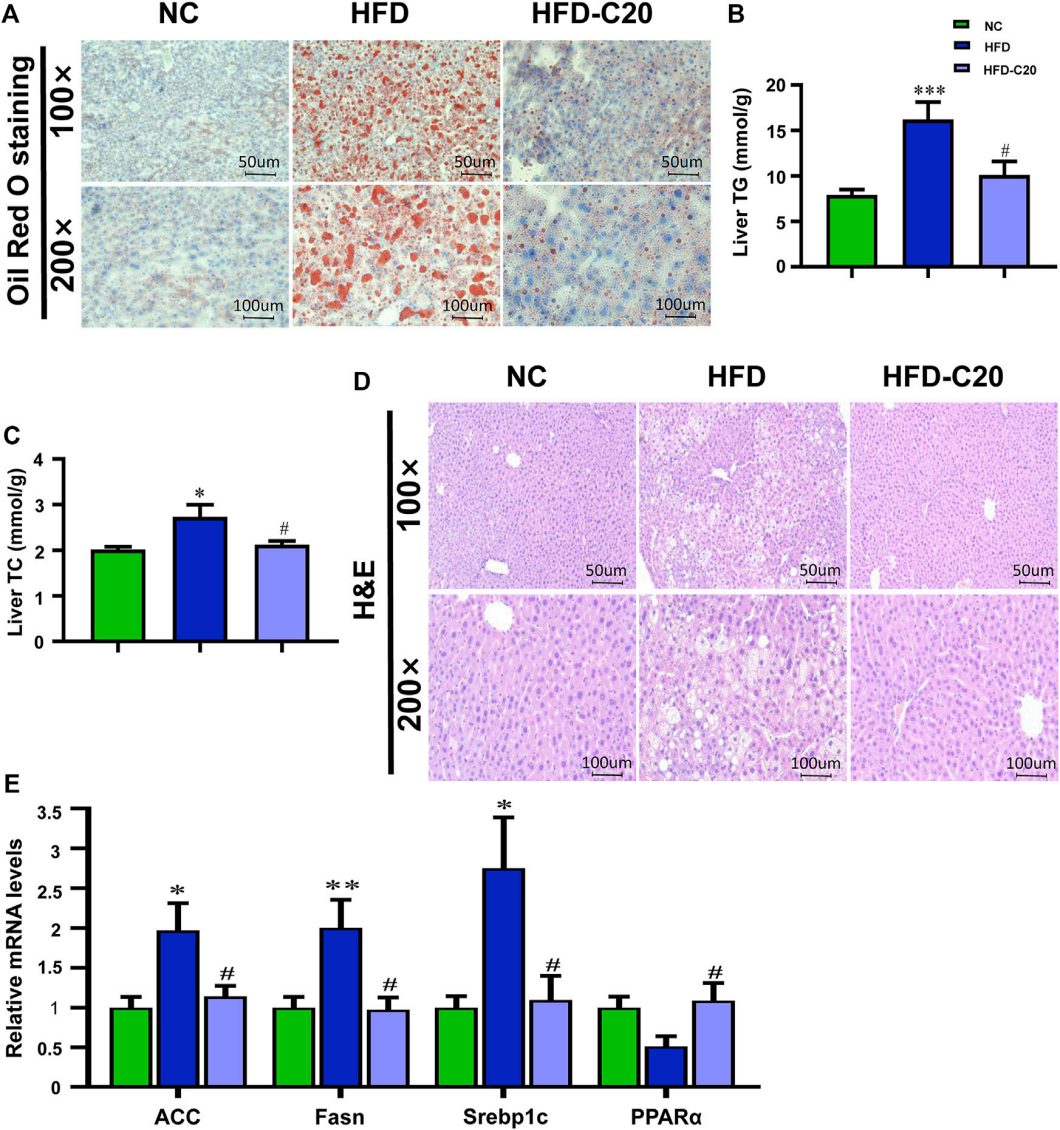
Crocin-I alleviates lipid accumulation in the liver induced by high-fat diet. **(A)** Lipid accumulation in liver tissue revealed by Oil Red O staining. **(B)** Levels of TG in the liver. **(C)** Levels of TC in the liver. **(D)** H&E staining of liver tissue. **(E)** mRNA expression of genes related to fatty acid synthesis. *n* = 8 per group. Values represent mean ± SEM. *p < 0.05 and **p < 0.01, compared with control group; ^#^ *p* < 0.05 compared with HFD group.

To examine the effect of crocin-I on liver physiology in mice in the HFD group, we analyzed lipid accumulation in the liver. Oil Red O staining showed increased liver lipid accumulation in the HFD group, but this effect was reversed after crocin-I treatment (Figure 2A). Liver TG and TC were significantly increased in the HFD group and were almost restored to the levels seen in controls after crocin-I treatment (Figures 2B,C). As depicted in Figure 2D, there were a large number of lipid droplets in hepatocytes in the HFD group, but the number decreased after crocin-I treatment. We next clarified that crocin-I affected liver metabolism and evaluated the expression of lipid-related genes using RT-qPCR. The mRNA expression of acetyl-CoA carboxylase (*ACC*), *Fasn,* and *Srebp1c* increased in the HFD group compared with controls (Figure 2E). Meanwhile, mRNA expression of *PPARα* was decreased, although not significantly (Figure 2E). Crocin-I reversed the changes of all these genes in the HFD group (Figure 2E). Our discoveries suggest that crocin-I rescued lipid metabolic disorders in HFD mice by counteracting the expression of genes controlling the fatty acid synthesis.

### Crocin-I Inhibited Intestinal Inflammation and Improved Intestinal Barrier Functions in HFD Mice

**FIGURE 3:**
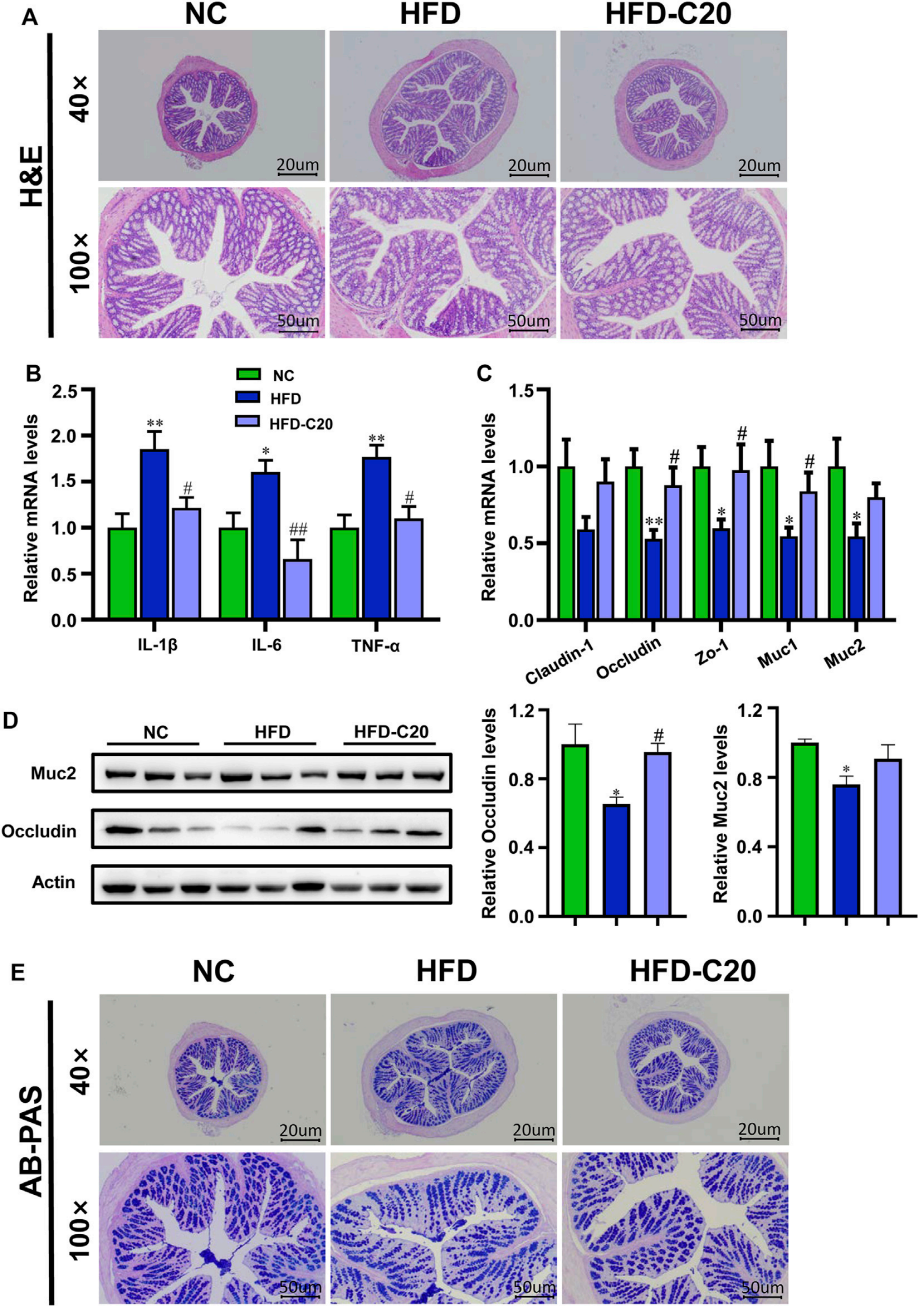
Crocin-I inhibited the low-grade inflammation and improved the intestinal barrier function in HFD mice. **(A)** H&E staining of the colon tissue. **(B)** The relative mRNA expression of inflammatory cytokines and **(C)** Tight junction proteins and mucin secretion in the colon tissue (*n* = 8). **(D)** The relative protein expression of. junction proteins and mucin secretion in the colon tissue (*n* = 3). **(E)** AB-PAS staining of the colon tissue. Values represent mean ± SEM. *p < 0.05 and **p < 0.01, compared with control group; ^#^ *p* < 0.05 and ^##^ *p* < 0.01, compared with HFD group.

H&E staining showed no obvious alterations to colonic mucosa villi, or epithelial, crypts or goblet cells in either the HFD group or the crocin-I treated group (Figure 3A). However, we wanted to examine the effects of crocin-I on the HFD-induced phenotype (i.e., intestinal inflammation) at a molecular level. The levels of inflammatory cytokines (including IL-1β, IL-6, and TNF-α) were measured in colon tissues. Compared with the NC group, the HFD group showed increased mRNA expressions of these inflammatory cytokines (Figure 3B), while crocin-I reduced mRNA expressions of *IL-1β, IL-6*, and *TNF-α* in colon tissues (Figure 3B). Furthermore, we tested the effect of crocin-I on the intestinal barrier by detecting transcription levels of genes related to tight junction proteins. The results showed decreased mRNA expression of *Occludin* and *Zo-1* in the HFD group when compared with the controls (Figure 3C), but these mRNA levels were restored by crocin-I treatment. However, no differences were seen in *Claudin-1* mRNA expression amongst the three groups (Figure 3C). Western blot results showed that Occludin significantly decreased in the HFD group, and crocin-I treatment restored it to normal levels (Figure 3D). AB-PAS staining of the colon indicated that the secretion of mucin in HFD group was significantly decreased relative to the NC group (Figure 3E). We also explored the mRNA expression levels of genes that were correlated with mucin secretion in colon tissue. The *Muc1* and *Muc2* mRNA levels in the HFD group decreased compared with the NC group (Figure 3C), while treatment with crocin-I increased *Muc1* and *Muc2* mRNA expression (Figure 3C). Muc2 protein levels were also significantly reduced in the HFD group (Figure 3D) and were not restored by crocin-I treatment. These results indicate that HFD may damage the integrity of the intestinal barrier and that treatment with crocin-I can repair it.

### Crocin-I Altered the Compositions of Gut Microbiota in HFD Mice

**FIGURE 4:**
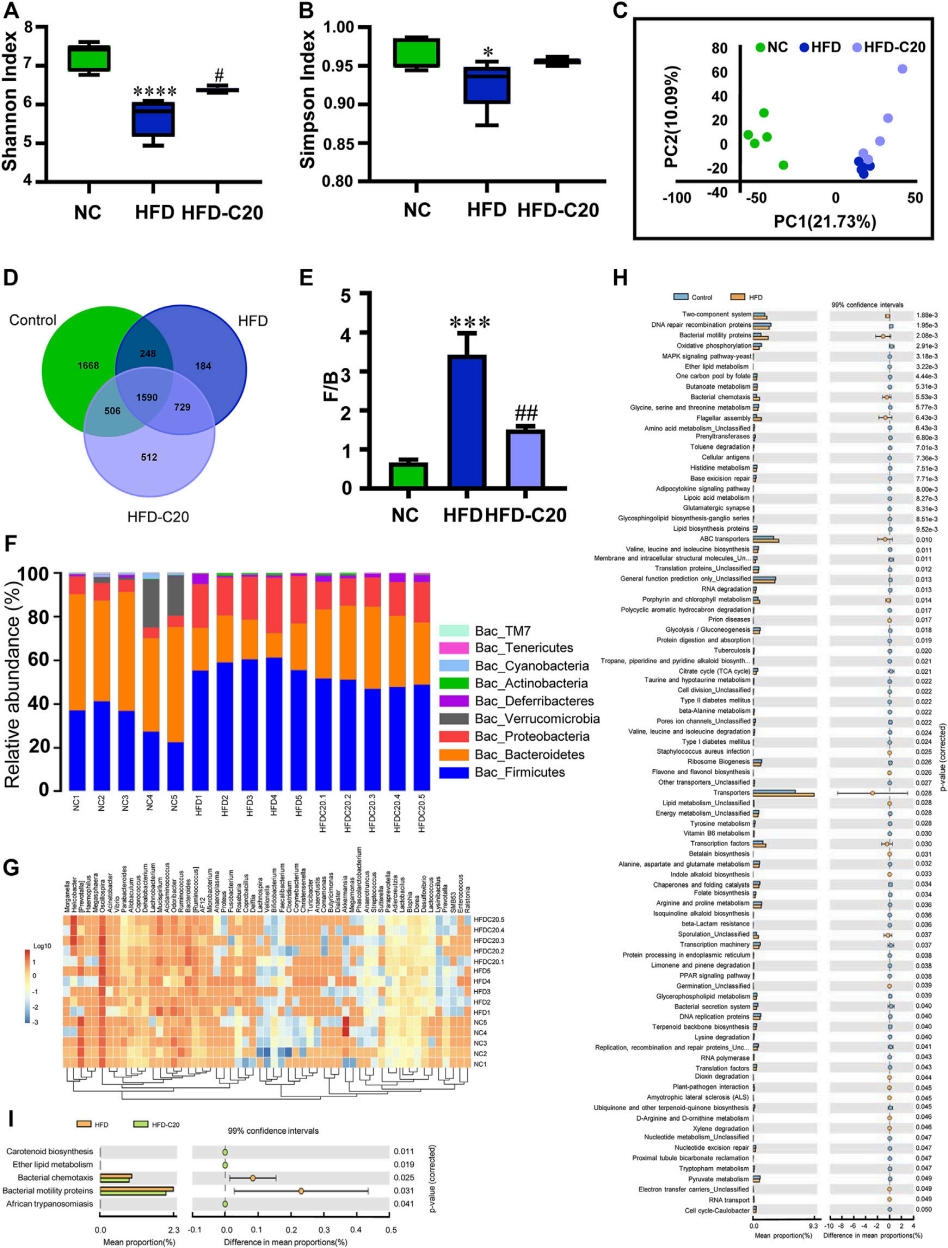
Crocin-I altered the gut microbiota composition of HFD mice. Alpha-diversity **(A)** Shannon index and **(B)** Simpson index (*n* = 5). **(C)** The PCoA score plot of the metabolic profiling from the different groups. The green dots represent the control group, the blue dots represent the HFD group, and the purple dots represent the HFD-C20 group (*n* = 5). **(D)** Venn diagrams showing the numbers of unique and shared OTUs in the different groups. The numbers shown where circles overlap are the number of OTUs shared by groups. The numbers adjacent to the numbers of shared OTUs indicate the number of unique OTUs. **(E)** Ratio of Firmicutes/Bacteroidetes which is F/B. **(F)** Relative abundances of the cecal microbiota at the phylum level. **(G)** Community structure differences and cluster analysis heat map. **(H)** Abundances of KEGG pathways in the functional prediction between NC and HFD. **(I)** Abundances of KEGG pathways in the functional prediction between HFD and HFD-C20. Values represent mean ± SEM. *p < 0.05 and **p < 0.01, compared with control group; ^#^ *p* < 0.05 and ^##^ *p* < 0.01, compared with HFD group.

We used high-throughput sequencing of 16S rRNA to evaluate the effects of crocin-I on the gut microbiota compositions of HFD mice. According to the Shannon and Simpson index, microbiota diversity was lower in the HFD group compared with controls (Figures 4A,B), but this diversity was restored with crocin-I treatment. In addition, unifrac-based PCoA demonstrated that microbiota composition exhibited distinct clustering in all three groups (Figure 4C). A total of 4012 OTUs in the NC group, 2751 OTUs in the HFD group, and 3337 OUTs in the HFD-C20 group were displayed in a Venn diagram, with 1590 OTUs shared (Figure 4D). At the phylum level, compared with the NC group, cecum *Bacteroidetes*, *Cyanobacteria*, and *TM7* abundances were significantly decreased in the HFD group, while *Proteobacteria* and *Firmicutes* abundances were significantly increased (*p* < 0.05, Figure 4F). Additionally, the abundances of *Defferribacteres* and *Actinobacteria* were increased, and *Verrucomicrobia* and *Tenericutes* levels were reduced in HFD mice, but not to a significant degree (Figure 4F). Strikingly, treatment with crocin-I reversed the changes in *Bacteroidetes*, *Firmicutes,* and *Proteobacteria* in HFD group mice (Figure 4F; Heat Map: Figure 4G). The F/B ratio was increased in HFD mice but decreased after crocin-I treatment (Figure 4E).

Next, metabolic pathways related to differential metabolites were tested using the KEGG database. High-fat feeding changed many metabolic pathways, including ether lipid metabolism, butanoate metabolism, glycolysis/gluconeogenesis, valine, leucine and isoleucine degradation, and glycerophospholipid metabolism (Figure 4H). Crocin-I administration changed the metabolic pathways related to carotenoid biosynthesis, ether lipid metabolism, bacterial chemotaxis, and the synthesis of bacterial motility proteins (Figure 4I).

### Crocin-I Improved SCFAs Level in the Feces of HFD Mice

**FIGURE 5:**
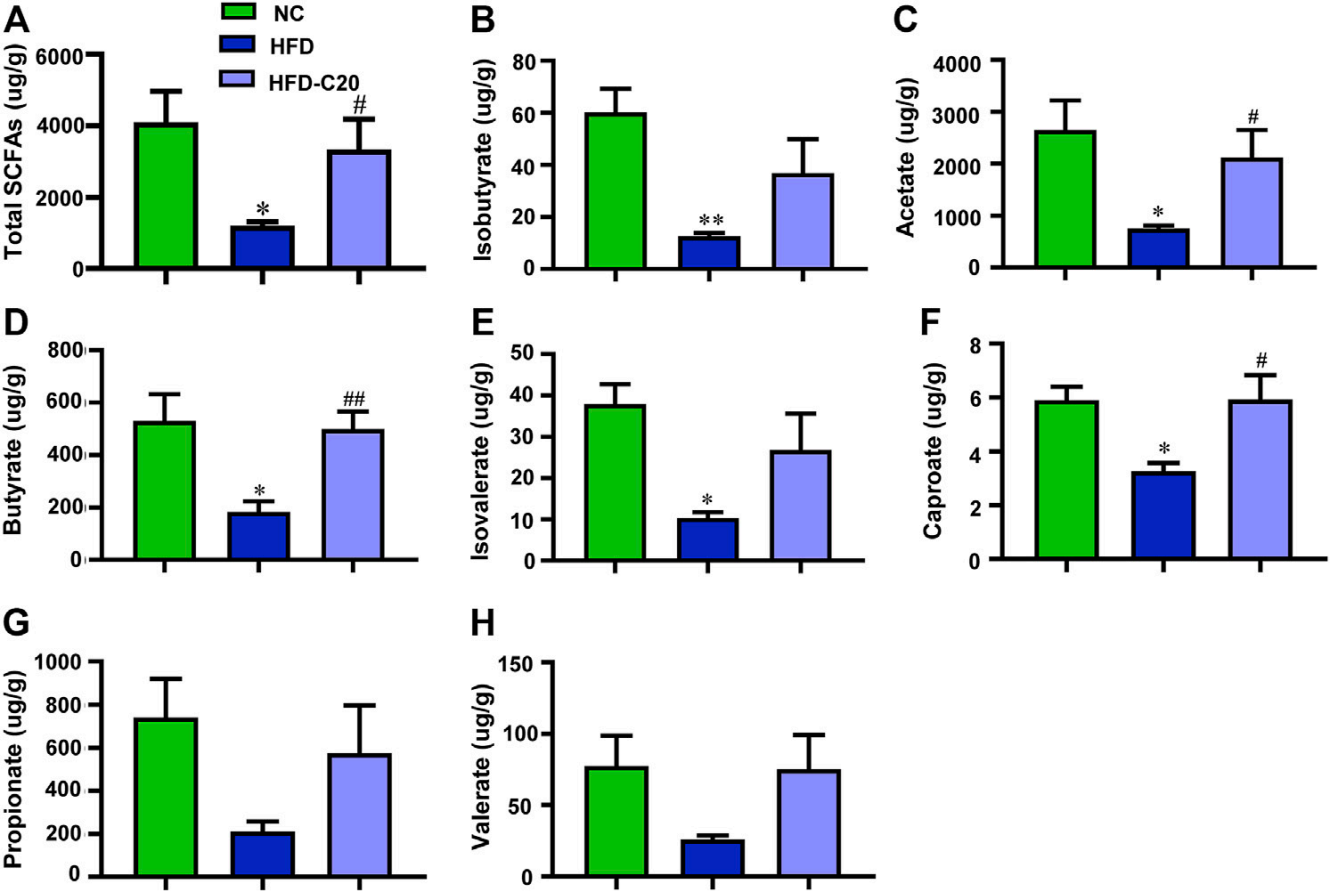
Crocin-I improved the SCFAs level in the feces of HFD mice. **(A)** Total SCFAs, **(B)** isobutyrate, **(C)** acetate, **(D)** butyrate, **(E)** isovalerate, **(F)** caproate, **(G)** propionate, and **(H)** valerate. *n* = 4 each group. Values represent mean ± SEM. *p < 0.05 and **p < 0.01, compared with control group; ^#^ *p* < 0.05 and ^##^ *p* < 0.01, compared with HFD group.

As shown in Figure 5, the total SCFAs level decreased in the HFD group when compared with the control group but this trend was rescued after crocin-I treatment (Figure 5A). Isobutyrate levels were reduced in the feces of the HFD group but treatment with crocin-I increased fecal isobutyrate levels (Figure 5B). The levels of acetate (Figure 5C), butyrate (Figure 5D), isovalerate (Figure 5E), caproate (Figure 5F), propionate (Figure 5G), and valerate (Figure 5H) had similar trends. Taken together, these results suggest that crocin-I could restore the reduction of fecal SCFAs in HFD-induced obese mice.

## Discussion

Crocin-I has a variety of pharmacological properties, including anti-inflammatory properties, lipid reduction abilities, and anti-cancer properties (Festuccia et al., 2014; Elsherbiny et al., 2016). It can also affect metabolism by regulating the composition of intestinal microbiota (Rayalam et al., 2008). Accumulating evidence suggests that gut microbiota contributes to the development of obesity and related diseases (DiBaise et al., 2008). Here, we report that crocin-I influences the composition of gut microbiota and lipid metabolism in high-fat-fed mice, thereby alleviating obesity.

In this study, we noted lipid accumulation in hepatocytes in HFD-treated mice. However, after crocin-I treatment, liver TG and TC contents were significantly reduced, and lipid synthesis and metabolic pathways were inhibited. This evidence suggests that crocin-I helped alleviate lipid accumulation. This conclusion is supported by our previous article (Xie et al., 2019) and by other findings that suggest crocin-I has lipid-lowering effects (Peyravi et al., 2020). Additionally, ACC, which is regulated by Srebp1c, catalyzes the carboxylation of acetyl CoA (and thus the synthesis of malonyl-CoA) (Yan et al., 2017; Pereira et al., 2019). Malonyl-CoA is an essential component for the *de novo* synthesis of fatty acids. Increased *ACC* mRNA expression can promote the large-scale synthesis of malonyl-CoA, while Fasn catalyzes the combination of acetyl-CoA and malonyl-CoA to form long-chain fatty acids (Nowacka-Woszuk et al., 2017). Changes in glycolysis/gluconeogenesis pathways can lead to glycerol synthesis, which eventually results in the accumulation of TG in the liver (Tian et al., 2022). These observations explain our findings that HFDs induced significant increases in *Fasn* and *ACC* mRNA expression, leading to hepatic lipid accumulation in mice. PPARα plays an important role in increasing fatty acid oxidation and lipid degradation to reduce lipid accumulation (Ji et al., 2021). Our findings of decreased *ACC*, *Fasn* and *Srebp1c* mRNA expression and increased *PPARα* mRNA expression in crocin-I treated mice suggest that crocin-I may play an important role in alleviating lipid metabolism disorders in the liver. In addition, previous studies have shown that SCFAs such as acetate and propionate can reduce the expression of lipid synthesis-related genes such as *Fasn* and *ACC*, and thus decrease adipogenesis (Jiao et al., 2021). This result may explain our findings that crocin-I could reduce lipid accumulation in mice to some extent by adjusting the SCFA content. However, this mechanism needs to be further studied.

The intestinal epithelium is the largest barrier against the external environment and is responsible for nutrition exchange. In addition, tight junction proteins are critical to the formation of the barrier. The protective effect of the barrier is enhanced by the presence of both the mucous layer and host-generated immune factors (Everard et al., 2013). Accumulating evidence has demonstrated that HFDs can down-regulate the expression of tight junction proteins and disrupt tight junction functioning, which results in many adverse effects on intestinal barrier integrity (Rohr et al., 2020). These findings are supported by our observations of decreased mRNA expression of tight junction proteins including *ZO-1* and *Occluding*. In addition, HFD mice have been shown to have increased intestinal permeability, along with increased expression of pro-inflammatory cytokines (Boscaini et al., 2021), which is consistent with our findings of damaged intestinal barriers in HFD mice. Previous studies have shown that the destruction of the mucus layer of the intestine increases its permeability (Wei et al., 2012). Similarly, our current study revealed that the expression levels of the mucus secretion-related genes *Muc1* and *Muc2* were also decreased, which may be related to increased intestinal permeability. Notably, our current study revealed that crocin-I decreased inflammatory factors and alleviated the inflammatory responses of colon tissue and recovered intestinal barrier functioning through up-regulation of tight junction proteins, all of which are consistent with our previous results (Xiao et al., 2020). These results also suggest that crocin-I may change the structure and composition of gut microbiota in high-fat diet-induced obese mice; however, this concept needs to be further studied.

Increasing evidence suggests that gut microbiota play a key role in modulating host immunity and metabolism (Vaarala et al., 2008). The F/B ratio has been linked to obesity and metabolic diseases (Hung et al., 2021), and its elevation should be linked to better absorption of energy via gut microbiota, and increased lipid synthesis, as well as lead to lipid metabolism disorders (Ge et al., 2020). Additionally, a previous study has indicated that the increased abundance of *Proteobacteria* was related to dysbiosis of gut microbiota (Shin et al., 2015). These previous studies support our observations that HFD significantly increased the F/B ratio and *Proteobacteria* abundance, while administration of crocin-I could reverse these tendencies. This suggests that crocin-I can alleviate obesity symptoms. In addition, our PCoA findings demonstrated that HFD group microbiota communities were compositionally distinct when compared to the control group and that these changes were not affected by crocin-I. Following an HFD, the microbial community’s alpha diversity was significantly reduced. However, crocin-I treatment did not affect these changes. Additionally, we did not find that crocin-I had any effects on the microbiota composition in the HFD group at the genus level. Given that environmental changes have a significant impact on the composition of intestinal microbes, crocin’s relationship with intestinal microbiota may be affected by different feeding conditions (Wang R. et al., 2019). Taken together, these results confirm that crocin-I may alleviate gut microbiota disorders in HFD mice.

SCFAs are the primary end-products of the saccharolytic fermentation of non-digestible carbohydrates that are not digested or absorbed in the small intestine (Flint et al., 2015). They are generated as a result of the complex interplay between nutrition and gut microbiota within the gut environment. Their major products are acetate, butyrate, propionate, and formate (Morrison and Preston, 2016). In our study, HFD mice showed decreased acetate, butyrate, caproate, and propionate levels. However, these levels were restored by crocin-I. This may be due to an increased abundance of *Bacteroidetes,* and a reduced abundance of *Firmicutes* and *Proteobacteria*. These findings can be explained by previous results showing how *Bacteroidetes* were directly linked to the production of isobutyrate, butyrate, isovalerate, and caproate (Ito et al., 1997) and that *Firmicutes* and *Proteobacteria* were negatively associated with the production of acetate, butyrate, and isovalerate (Wang M. et al., 2019). Furthermore, other studies have shown that SCFAs have important regulatory effects on metabolic and immune responses (Smith et al., 2013). Acetate has been linked to the suppression of inflammatory responses via the regulation of a variety of immune cells (Lei et al., 2021). Central administration of butyrate can improve the intestinal barrier, which reduces the permeability of the colon (Okumura et al., 2021). Additionally, butyrate and propionate have important effects on the regulation of immune and inflammatory responses (Liu et al., 2018; Duscha et al., 2020). These results can also explain our findings that crocin-I treated mice with improved SCFA content also had improved intestinal inflammation and intestinal barrier function. Taken together, our results suggest that crocin-I may affect intestinal inflammation and intestinal barrier function by regulating SCFA content.

In conclusion, our research shows that crocin-I can reverse the effects of high-fat feeding on lipid synthesis in the liver and that it improves impaired lipid-related metabolic pathways, intestinal microbial disorders, and disrupted SCFA levels. Crocin-I also alleviates inflammation in mice, which helps with lipid metabolism and reduces lipid content. These findings provide important data to support the use of crocin-I as a drug for the treatment of obesity.

## Data Availability

The raw data supporting the conclusions of this article will be made available by the authors, without undue reservation.

## Abbreviations

ACC, acetyl-CoA carboxylase; Srebp1c, sterol regulatory element binding protein 1c; Fasn, fatty acid synthase; ANOVA, analysis of variance; GC-MS, gas chromatography–mass spectrometry; HFD, high-fat diet-induced; H&E, hematoxylin and eosin; IPGTT, intraperitoneal glucose tolerance test; RT-qPCR, real-time quantitative polymerase chain reaction; SCFA, short-chain fatty acid.
