# Supplementary figures and images for: Crocin-I Protects Against High-Fat Diet-Induced Obesity via Modulation of Gut Microbiota and Intestinal Inflammation in Mice
Source: Front Pharmacol. 2022 Aug 11;13:894089. doi: 10.3389/fphar.2022.894089 (PMC9403484; doi:10.3389/fphar.2022.894089)

**2 A**

**NC 100×**

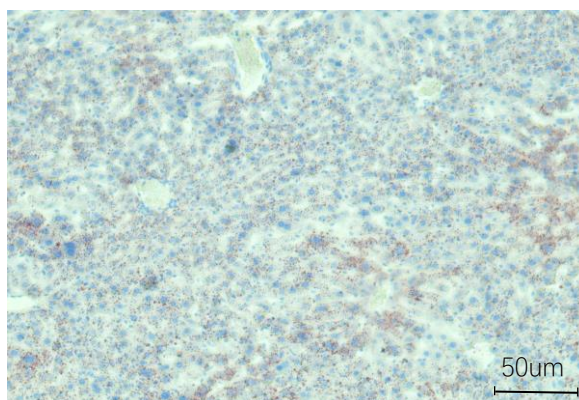

**HFD 100×**

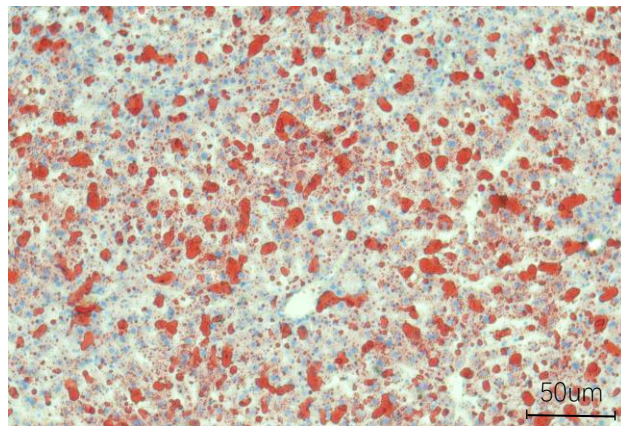

**HFD-C20 100×**

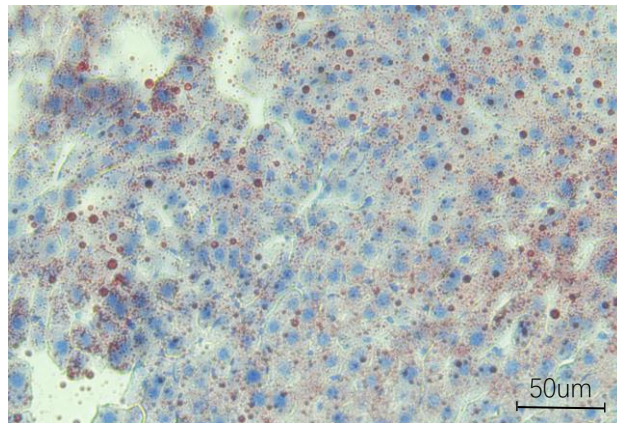

**NC 200×**

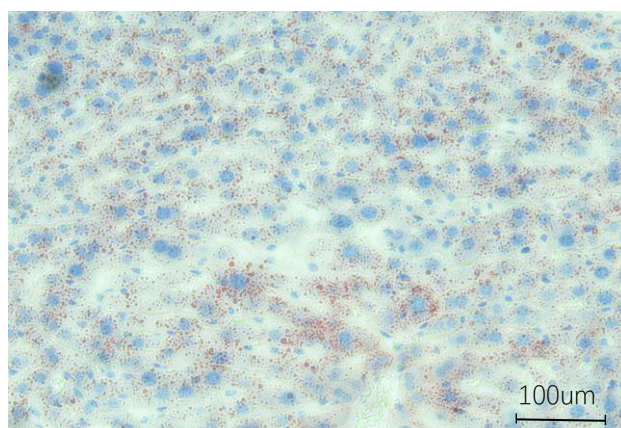

**HFD 200×**

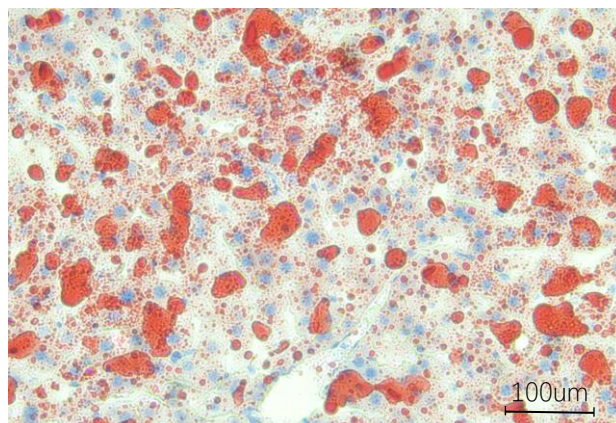

**HFD-C20 200×**

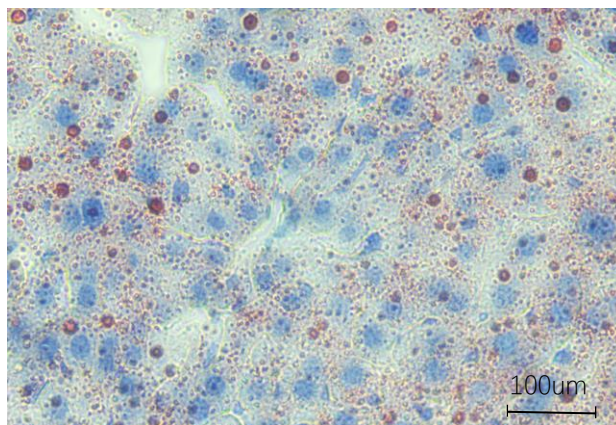

**2 C**

**NC 100×**

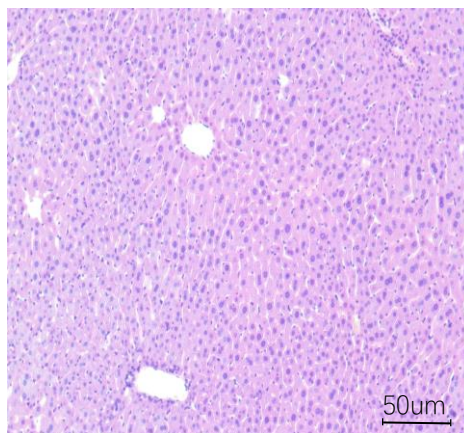

**HFD 100×**

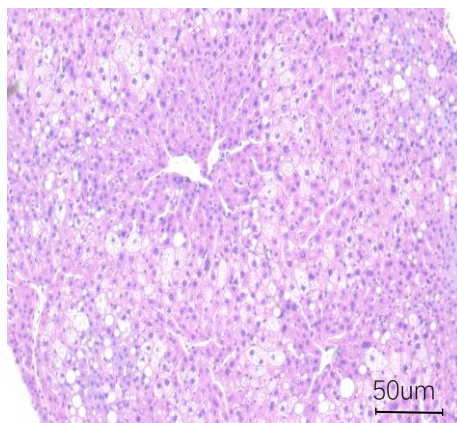

**HFD-C20 100×**

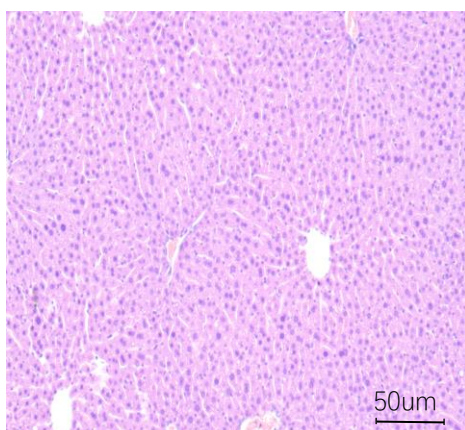

**NC 200×**

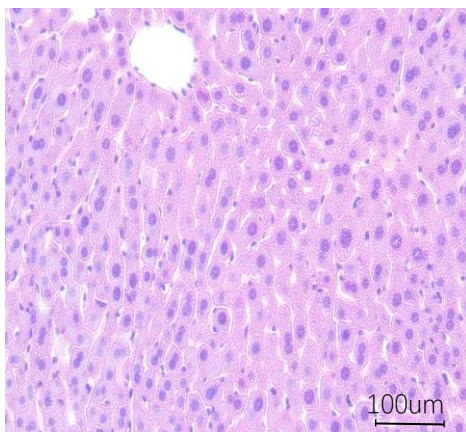

**HFD 200×**

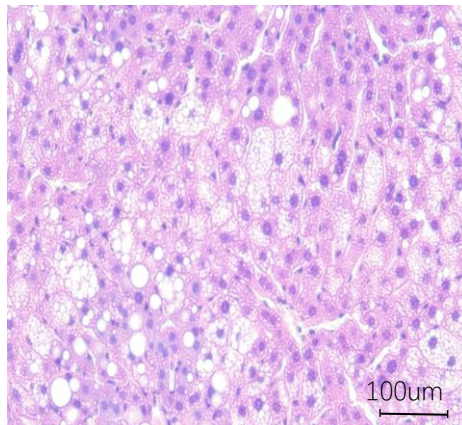

**HFD-C20 200×**

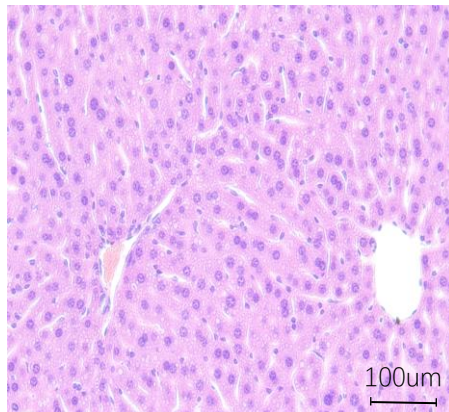

Supplement: Supplementary file 1 [file DataSheet7.PDF]

**NC 100×**

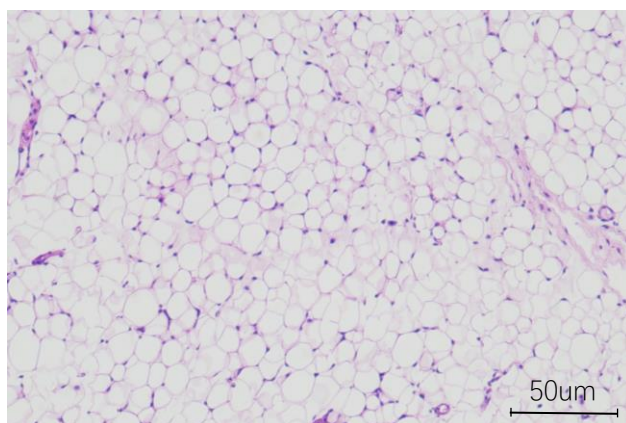

**HFD 100×**

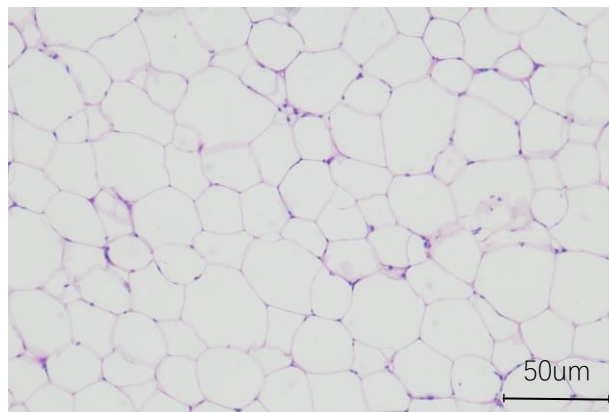

**HFD-C20 100×**

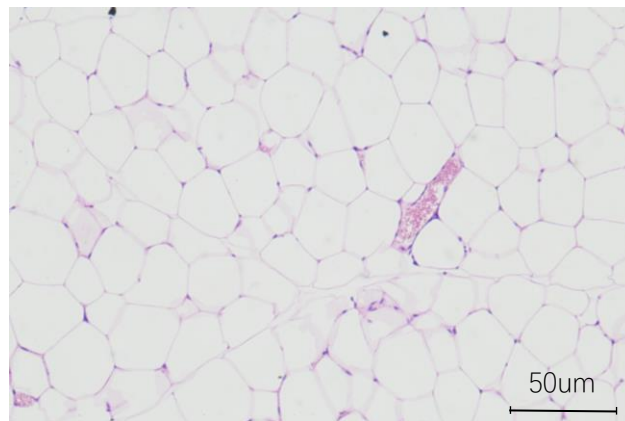

**NC 200×**

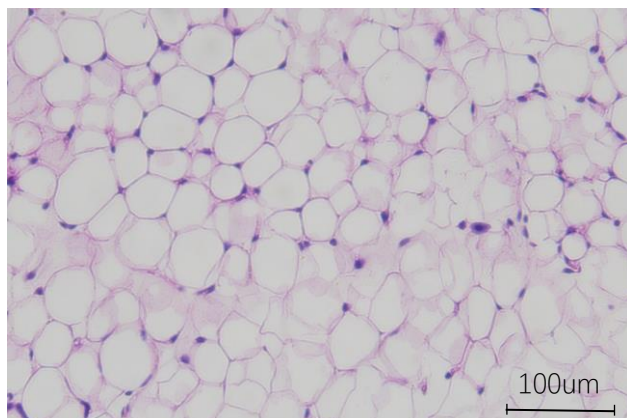

**HFD 200×**

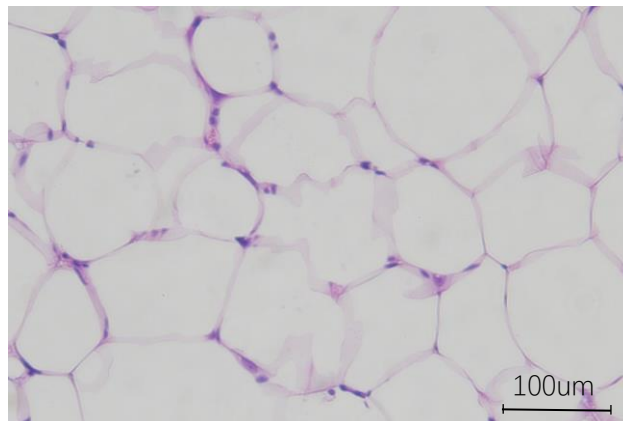

**HFD-C20 200×**

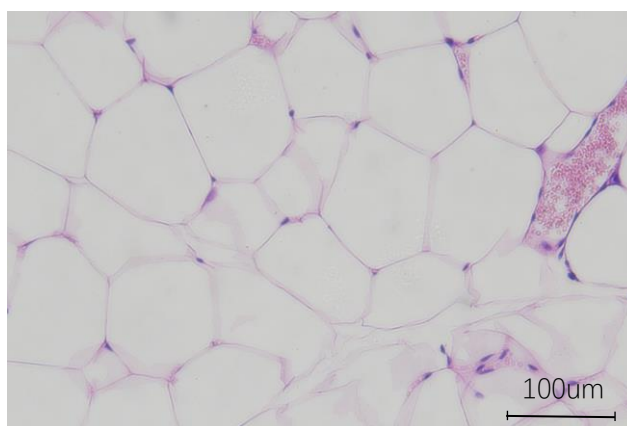

Supplement: Supplementary file 2 [file DataSheet6.PDF]

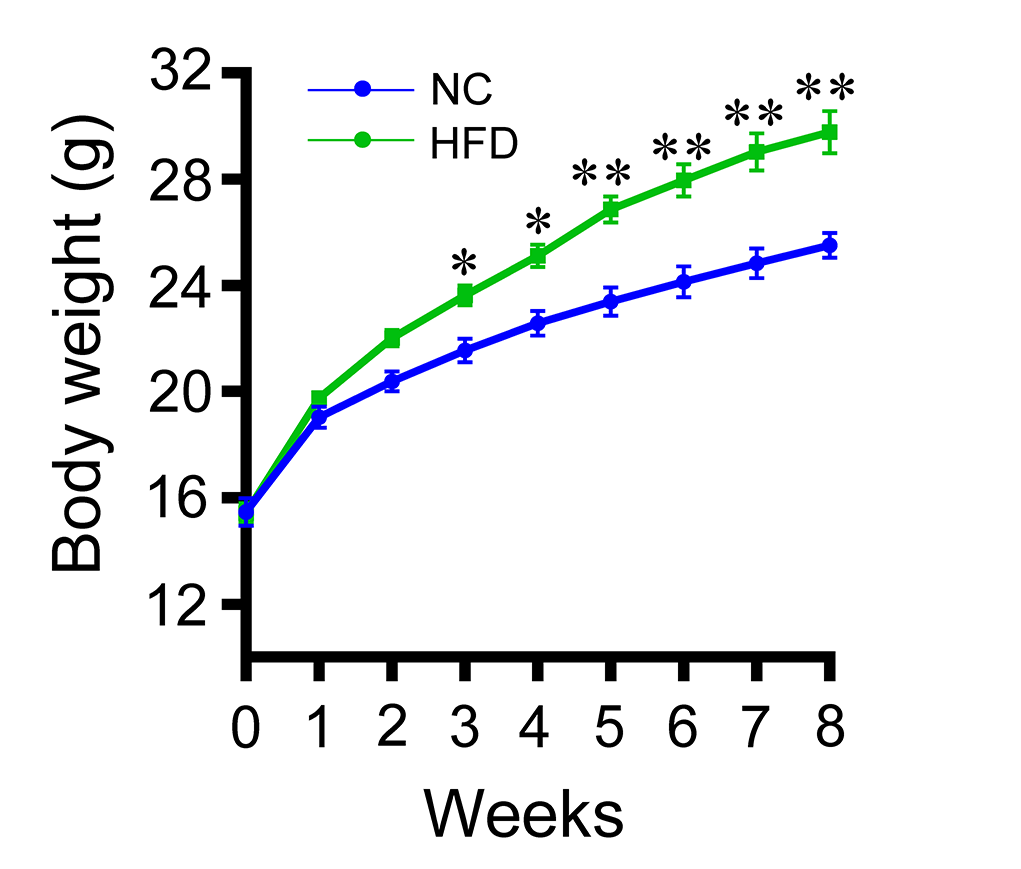

Supplement: Supplementary file 5 [file Image2.TIF]

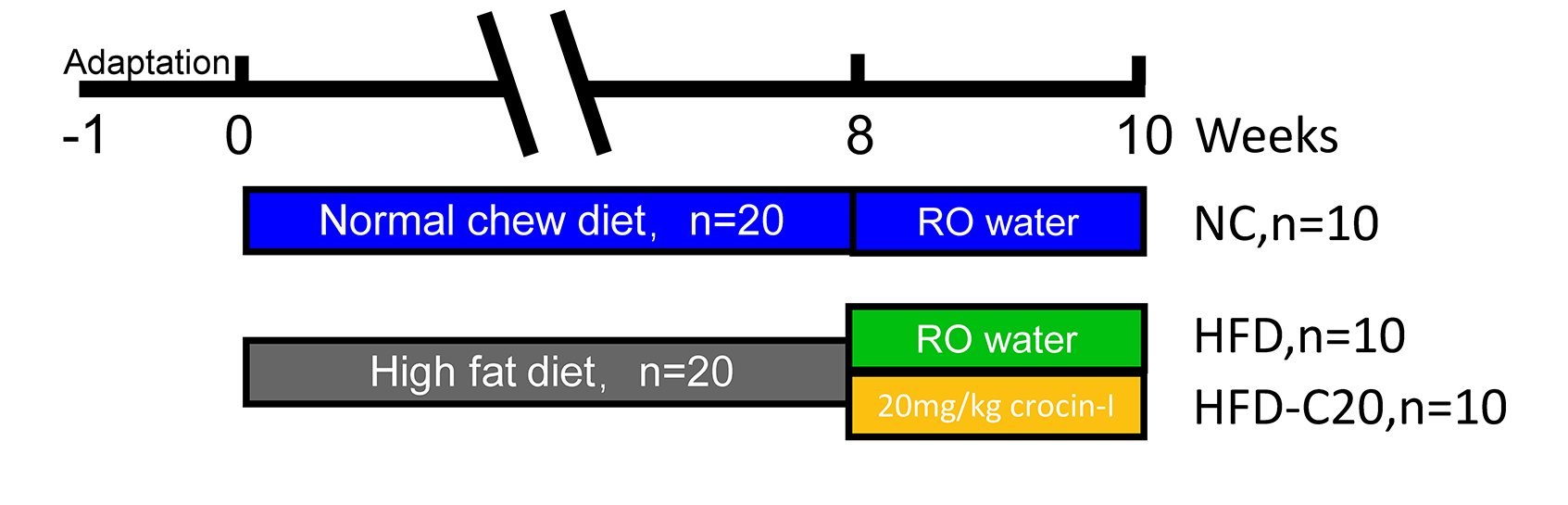

Supplement: Supplementary file 6 [file Image1.TIF]

**3 A**

**NC 40×**

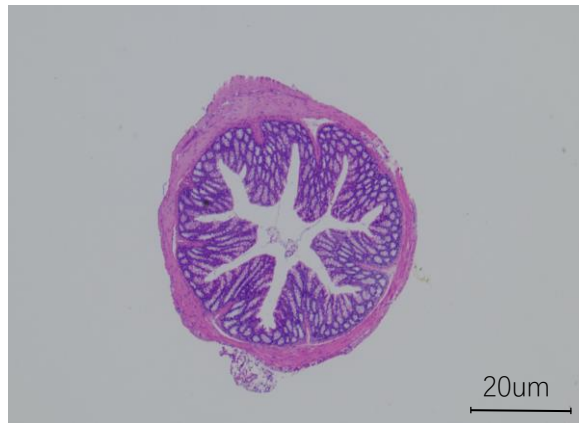

**HFD 40×**

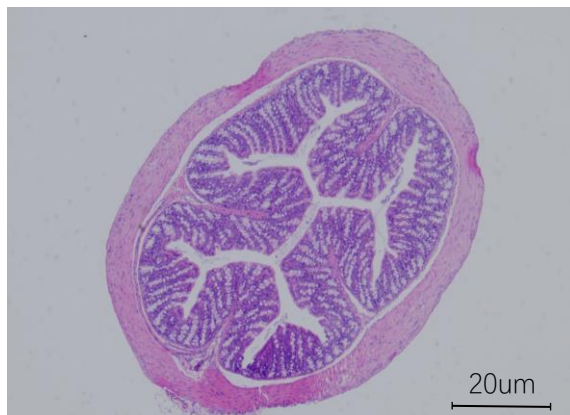

**HFD-C20 40×**

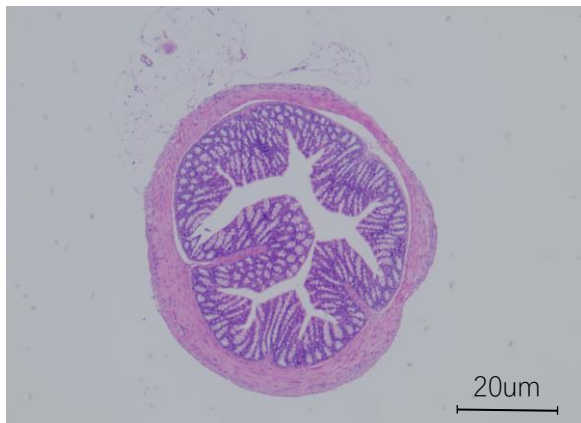

NC 100×

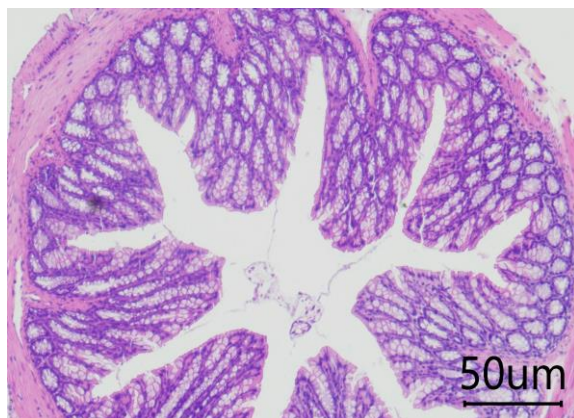

**HFD 100×**

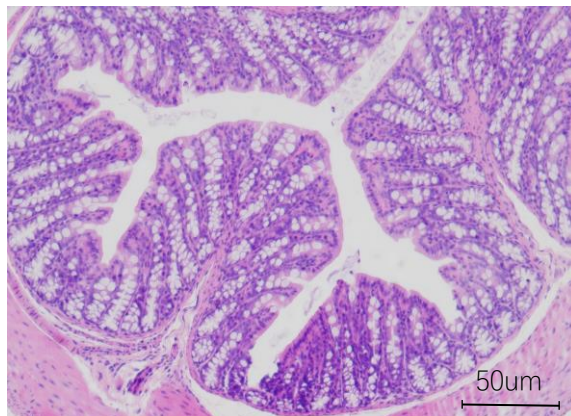

**HFD-C20 100×**

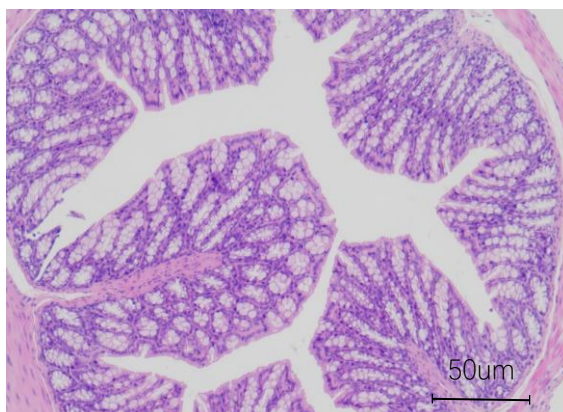

**3 D**

**NC 40×**

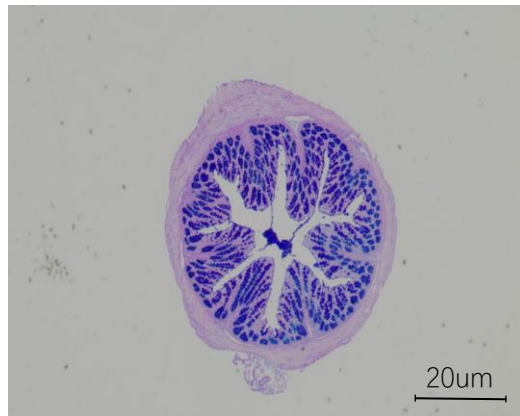

**HFD 40×**

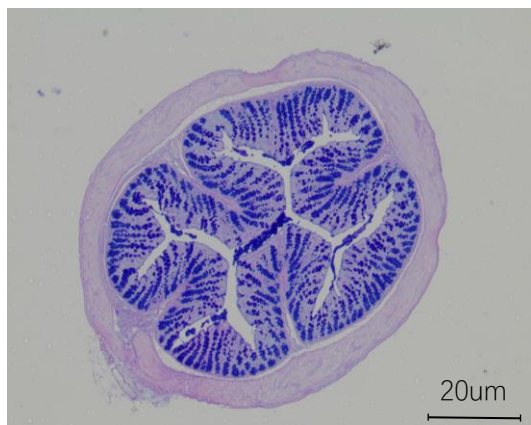

**HFD-C20 40×**

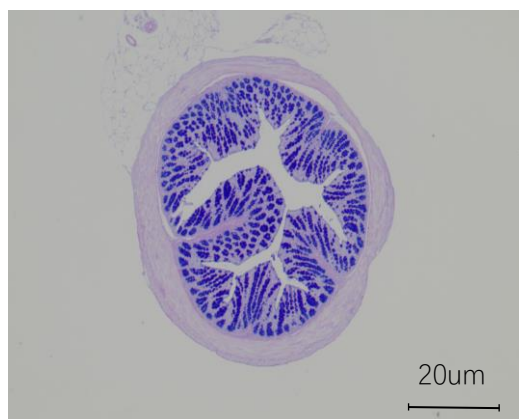

NC 100×

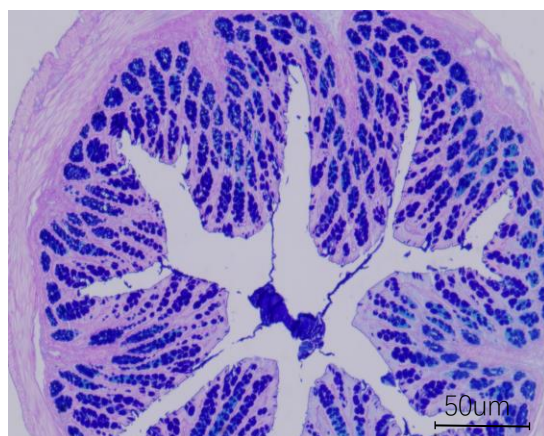

**HFD 100×**

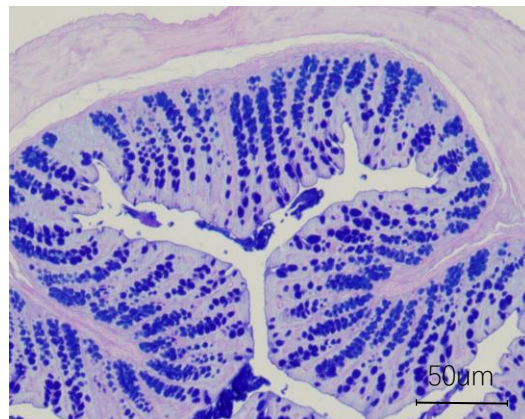

**HFD-C20 100×**

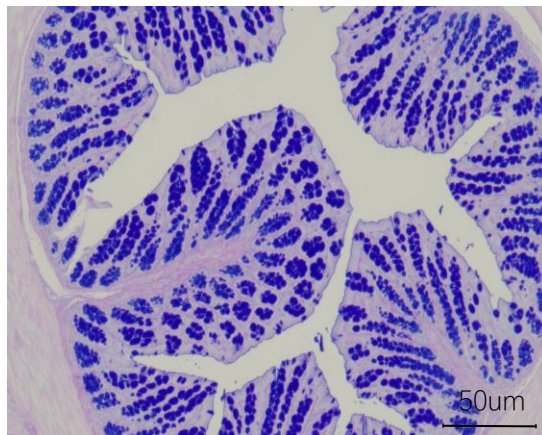

Supplement: Supplementary file 8 [file DataSheet8.PDF]
