# Supplementary material for: Crocin-I Protects Against High-Fat Diet-Induced Obesity via Modulation of Gut Microbiota and Intestinal Inflammation in Mice
Source: Front Pharmacol. 2022 Aug 11;13:894089. doi: 10.3389/fphar.2022.894089 (PMC9403484; doi:10.3389/fphar.2022.894089)
Supplement: Supplementary file 3 [file Table1.DOCX]

**Table S1** Sequences of primer pairs used in the real-time quantitative PCR reactions

| Gene name |  | Primer sequence |
| --- | --- | --- |
| Srebp-1c  TNF-α | Sterol regulatory element binding transcription factor 1  Tumor necrosis factor α | Forward 5’- TGACCCGGCTATTCCGTGA-3’  Reverse 5’- CTGGGCTGAGCAATACAGTTC-3’  Forward 5’-GTCTACTGAACTTCGGGGTGAT-3’  Reverse 5’-GGCTACAGGCTTGTCACTCG-3’ |
| Fasn | [Fatty acid synthase](https://www.ncbi.nlm.nih.gov/gene/2194) | Forward 5’- GGAGGTGGTGATAGCCGGTAT -3’  Reverse 5’- TGGGTAATCCATAGAGCCCAG-3’ |
| TLR4 | Toll-like receptor 4 | Forward 5’- ATGGCATGGCTTACACCACC -3’  Reverse 5’- GAGGCCAATTTTGTCTCCACA -3’ |
| ACC | Acetyl-CoAcarboxylase | Forward 5’- TGCAAACACCCTCCTTGTGT -3’  Reverse 5’- GGTCTTCGAGTGGATTGGCA-3’ |
| IL-6 | Interleukin 6 | Forward 5’-CTCTGCAAGAGACTTCCATCC-3’  Reverse 5’-GAATTGCCATTGCACAACTC-3’ |
| MUC1 | Mucin 1 | Forward 5’- GGCATTCGGGCTCCTTTCTT-3’  Reverse 5’- TGGAGTGGTAGTCGATGCTAAG-3’ |
| MUC2 | Mucin 2 | Forward 5’- ACACTCAGCACACCAACCAA-3’  Reverse 5’- CACAGCCACCAGGTCTCATT-3’ |
| Cpt1 | Carnitine palmitoyltransferase 1a | Forward 5’- CTCCGCCTGAGCCATGAAG-3’  Reverse 5’- CACCAGTGATGATGCCATTCT-3’ |
| PPARα | Peroxisome proliferator activated receptor alpha | Forward 5’- AGAGCCCCATCTGTCCTCTC-3’  Reverse 5’- ACTGGTAGTCTGCAAAACCAAA -3’ |
| GPX | Glutathione peroxidase 1 | Forward 5’- AGTCCACCGTGTATGCCTTCT-3’  Reverse 5’- GAGACGCGACATTCTCAATGA-3’ |
| Claudin1 | Claudin 1 | Forward 5’-CTGGAAGATGATGAGGTGCAGAA GA -3’  Reverse 5’- CCACTAATGTCGCCAGACCTGAA-3’ |
| Occludin | Occludin | Forward 5’-TGCTTCATCGCTTCCTTAGTAA-3’  Reverse 5’-GGGTTCACTCCCATTATGTACA-3’ |
| ZO-1 | Tight junction protein 1 | Forward 5’-CTGGTGAAGTCTCGGAAAAATG-3’  Reverse 5’-CATCTCTTGCTGCCAAACTATC-3’ |
| IL-1β | Interleukin 1β | Forward 5’-CCAACAAGTGATATTCTCCATGAG -3’  Reverse 5’-ACTCTGCAGACTCAAACTCCA-3’ |
| CAT | Catalase | Forward 5’- AGCGACCAGATGAAGCAGTG-3’  Reverse 5’- TCCGCTCTCTGTCAAAGTGTG -3’ |
| SOD2 | Superoxide dismutase 2 | Forward 5’- CAGACCTGCCTTACGACTATGG-3’  Reverse 5’- CTCGGTGGCGTTGAGATTGTT -3’ |
| β-actin | β-actin | Forward 5’- GGCTGTATTCCCCTCCATCG -3’  Reverse 5’- CCAGTTGGTAACAATGCCATGT -3’ |
| 16s |  | Forward 5’- ACTCCTACGGGAGGCAGCAG-3’  Reverse 5’- ATTACCGCGGCTGCTGG -3’ |
